# Evaluation of postoperative trismus in buccal versus lingual approach for impacted mandibular third molar removal

**DOI:** 10.6026/973206300211113

**Published:** 2025-05-31

**Authors:** Nitin Purohit, Dipanjal Saikia, Abhigyan Manas, Abdul Kalam Azad, Shibabrata Behera, Vaibhav Rai, Sai Kiran Bahadur, Varadharajula Venkata Ramaiah

**Affiliations:** 1 Department of Dentistry, District Hospital, Rudraprayag, Uttarakhand, India; 2 Department of Dentistry, Assam Medical College and Hospital, Dibrugarh, Assam, India; 3 Department of Oral and Maxillofacial Surgery, Faculty of Dental Sciences, Uttar Pradesh University of Medical Sciences, Saifai, Etawah, India; 4 Department of Oral and Maxillofacial Surgery, College of Dentistry, Qassim University, Saudi Arabia; 5 Department of Oral And Maxillofacial Surgery, Kalinga Institute of Dental Sciences, KIIT Deemed to be University, Patia, Bhubaneswar-751024, Odisha, India; 6 Department of Oral Medicine and Radiology, Universal College of Medical Sciences, Nepal, India; 7 Department of Prosthodontics, Springfield Dental, Springfield, Massachussets, USA; 8 Department of Public Health, College of Applied Medical Sciences, Qassim University, Buraydah 51452, PSBox 6666, Saudi Arabia

**Keywords:** Trismus, buccal approach, lingual flap, impacted third molar, ROC curve, odds ratio

## Abstract

Evaluation of trismus incidence and severity between buccal and lingual approaches in impacted mandibular third molar extractions is of interest. Hence, a prospective observational study was conducted with 100 patients divided into two groups: Group A (buccal approach) and Group B (lingual approach). Clinical parameters including inter incisal distance (IID), Visual Analog Scale (VAS) for pain, facial swelling and surgery duration were assessed preoperatively and postoperatively on Days 1, 3 and 7. The lingual approach to mandibular third molar extraction is connected with a greater chances of severe postoperative trismus compared to the buccal approach.

## Background:

Impacted mandibular third molars are among the most commonly encountered cases in oral and maxillofacial surgery, often requiring surgical intervention due to pain, pericoronitis, or associated pathology [1]. The technique adopted for their removal plays a crucial role in determining postoperative outcomes, mainly in terms of patient comfort and recovery. One of the most common and significant postoperative complications is trismus, which is characterized by a restricted ability to open the mouth [2]. This limitation can interfere with essential functions such as eating, speaking, maintaining oral hygiene and undergoing dental treatment, thereby considerably affecting a patient's quality of life during the recovery period [3]. Surgical manipulation, especially involving deeper tissue layers and proximity to the medial pterygoid muscle, can influence the severity of trismus [4]. Therefore, it is of interest to assess the incidence, severity and duration of postoperative trismus following the removal of impacted mandibular third molars using the buccal versus lingual surgical approach.

## Materials and Methods:

This prospective observational comparative study was conducted to assess the incidence and severity of postoperative trismus in patients undergoing surgical removal of impacted mandibular third molars using two different approaches-buccal and lingual. A total of 100 patients aged between 18 and 35 years were recruited and equally divided into two groups: Group A (n=50) underwent the buccal approach, while Group B (n=50) underwent the lingual approach. All participants presented with mesioangular impactions classified as Pell and Gregory Class I or II and Position A or B. Patients with systemic illnesses, temporomandibular joint disorders, a recent history of pericoronitis (within one month), prior use of analgesics or steroids and pregnant or lactating women were excluded from the study to eliminate confounding variables. The key clinical parameters assessed included interincisal distance (IID) measured in millimeters, pain intensity using the Visual Analog Scale (VAS, 0-10); facial swelling using standardized reference points and duration of surgery in minutes. Trismus was categorized into three grades based on IID measurements: mild (>35 mm), moderate (20-35 mm) and severe (<20 mm). These parameters were recorded preoperatively and postoperatively on Day 1, Day 3 and Day 7 to assess progression and recovery. These parameters-interincisal distance (IID), VAS for pain, facial swelling and surgery duration-were selected as they collectively reflect the functional limitation, inflammation and surgical trauma contributing to the severity and recovery of postoperative trismus. All data were compiled and analyzed using SPSS version 25. Descriptive statistics such as mean, standard deviation and frequency distribution were calculated. Group comparisons were conducted using the independent t-test or the Mann-Whitney U test depending on data normality, while the chi-square test was used for categorical variables. A Receiver Operating Characteristic (ROC) curve analysis was performed to determine the optimal cutoff value for IID in predicting severe trismus. Additionally, odds ratios were calculated to estimate the relative risk of severe trismus between the two approaches and multivariate logistic regression was employed to adjust for potential confounders and identify independent predictors of postoperative trismus.

## Results:

A total of 100 patients were evaluated, with 50 in each group. The mean duration of surgery was significantly shorter in the buccal approach group (Group A), averaging 28.5 ± 3.6 minutes, compared to 32.1 ± 4.1 minutes in the lingual approach group (Group B), with a statistically significant difference (p = 0.002). Preoperatively, the interincisal distance (IID) was comparable between both groups, measuring 43.5 ± 2.5 mm in Group A and 42.8 ± 2.3 mm in Group B (p = 0.140), indicating no significant baseline difference. However, a marked difference was observed in postoperative IID values. On postoperative Day 1, Group A recorded a mean IID of 26.3 ± 4.1 mm, whereas Group B showed a significantly lower mean of 20.5 ± 3.9 mm (p < 0.001), indicating more pronounced trismus in the lingual approach group. The trend continued on Day 3, with IID measuring 32.2 ± 3.8 mm in Group A versus 26.4 ± 4.2 mm in Group B (p < 0.001) and on Day 7, where Group A achieved near-complete recovery at 38.4 ± 3.1 mm compared to 34.1 ± 3.6 mm in Group B (p < 0.001).The incidence of severe trismus (IID < 20 mm) was also significantly higher in the lingual approach group, with 36% of patients affected, in contrast to only 10% in the buccal approach group (p < 0.01). The odds ratio (OR) for developing severe trismus in the lingual group compared to the buccal group was calculated to be 5.1, with a 95% confidence interval ranging from 2.0 to 12.8, suggesting that patients undergoing the lingual approach were over five times more likely to develop severe postoperative trismus (Table 1). Receiver Operating Characteristic (ROC) curve analysis was performed to determine a predictive threshold for IID in identifying severe trismus. The area under the curve (AUC) was 0.87 (95% CI: 0.79-0.94), indicating excellent diagnostic accuracy. The optimal cutoff value for IID on postoperative Day 1 was found to be 24 mm, yielding a sensitivity of 82% and specificity of 76%. Table 1 presents a comparative analysis of surgical time and trismus-related outcomes between the buccal and lingual approaches for mandibular third molar removal. The lingual approach showed significantly greater postoperative trismus and higher odds of severe limitation in mouth opening compared to the buccal approach. The ROC curve analysis indicates that an IID of 24 mm on Day 1 post-surgery is the optimal threshold for predicting severe trismus. With a sensitivity of 82% and specificity of 76%, this cut off effectively distinguishes between mild and severe postoperative trismus (Table 2).

## Discussion:

Trismus, often referred to as "lockjaw," is a condition characterized by a reduced ability to open the mouth, typically caused by the restriction of normal jaw movement. It is a common complication following the surgical removal of impacted third molars, particularly mandibular molars. Trismus not only impairs the patient's ability to eat, speak and perform daily activities but also contributes significantly to postoperative discomfort and recovery time. The degree of trismus can vary, influenced by several factors, including surgical approach, tissue trauma, inflammation and individual healing responses [5, 6]. The surgical approach used in the extraction of impacted third molars is a key determinant of postoperative morbidity, including trismus. Two commonly employed approaches-buccal and lingual-are often debated in clinical practice for their differing impacts on recovery outcomes. Understanding how these approaches affect the severity of trismus and its underlying mechanisms is crucial for improving patient care and optimizing surgical strategies [6]. Trismus occurs as a result of various factors that impair the normal function of the masticatory muscles, particularly the lateral pterygoid and masseter muscles. During the surgical removal of impacted third molars, particularly in cases of mesioangular impactions, trauma to the muscle fibers and surrounding soft tissues can lead to muscle spasm, inflammation and fibrosis, which restricts the ability of the jaw to open [7]. In addition to muscle damage, inflammation in the surgical site-caused by tissue manipulation, retraction and bone removal-can increase the production of prostaglandins and other inflammatory mediators, leading to further muscle stiffness and swelling. This inflammatory response can result in both pain and mechanical restriction, which exacerbates trismus. Scar tissue formation and fibrosis that may develop as part of the healing process can also contribute to the persistence of trismus long after the initial surgical insult has resolved [8, 9]. The findings of this study highlight the significant impact of the surgical approach on postoperative trismus following the extraction of impacted mandibular third molars. The comparison between the buccal (Group A) and lingual (Group B) approaches provides valuable insights into the recovery process and the factors influencing the severity of trismus. The mean duration of surgery was significantly shorter in the buccal approach group (28.5 ± 3.6 minutes) compared to the lingual approach group (32.1 ± 4.1 minutes), which is consistent with the expectation that simpler, less invasive approaches require less time [10]. This finding aligns with previous research showing that prolonged surgical procedures generally result in more tissue trauma and longer recovery periods (Rizqiawan *et al.* 2022) [4]. The shorter duration of surgery in the buccal approach suggests that this approach may contribute to a reduced inflammatory response and, therefore, less postoperative trismus. The preoperative IID values in both groups were comparable (43.5 ± 2.5 mm in Group A and 42.8 ± 2.3 mm in Group B), which indicates that the baseline mouth opening was similar across groups. This is important because it eliminates any confounding effect from preexisting limitations in mouth opening. However, postoperative IID values demonstrated a clear difference. On Day 1, Group A showed a mean IID of 26.3 ± 4.1 mm, while Group B exhibited a significantly lower mean IID of 20.5 ± 3.9 mm (p < 0.001), indicating a greater degree of trismus in the lingual approach group. This trend persisted through Days 3 and 7, with Group A recovering more quickly than Group B. The data clearly show that the lingual approach resulted in a higher incidence of severe trismus, with 36% of patients in Group B developing severe trismus (IID < 20 mm), compared to only 10% in Group A (p < 0.01). The greater incidence of trismus in the lingual approach group may be attributed to the increased manipulation of surrounding tissues, including muscles like the lateral pterygoid, which can result in more significant postoperative swelling and pain [11]. The deeper dissection required for the lingual approach could also cause more trauma to the muscles of mastication, leading to greater muscle spasms and fibrosis, contributing to more pronounced trismus. According to Ge *et al.* (2016), their study concluded that among deeply or fully impacted mandibular third molars; the lingual position type is the most prevalent, followed by the central position type and then the buccal position type. The study also highlighted that the buccal and inferior inferior alveolar canal (IAC) courses are the most common characteristics in lingually positioned impacted molars. However, the choice of surgical approach varies based on the positioning of the molar. For lingually positioned molars, the study recommended the use of the lingual split technique, while the buccal approach was deemed the absolute indication for buccally positioned molars. As for centrally positioned third molars, the surgical approach is flexible, depending on individual variation [12]. The odds ratio (OR) for developing severe trismus in the lingual approach group was calculated to be 5.1, with a 95% confidence interval ranging from 2.0 to 12.8, indicating that patients undergoing the lingual approach were more than five times as likely to develop severe postoperative trismus compared to those undergoing the buccal approach. This finding underscores the increased risk associated with the lingual approach and highlights the importance of considering this factor when planning third molar extractions.

ROC curve analysis was employed to establish an optimal threshold for IID in predicting severe trismus. The area under the curve (AUC) was 0.87 (95% CI: 0.79-0.94), indicating excellent diagnostic accuracy for using IID as a predictive measure of trismus severity. The optimal cutoff for IID on postoperative Day 1 was identified as 24 mm, with a sensitivity of 82% and specificity of 76%. This suggests that a reduction in IID to below 24 mm on Day 1 is a strong indicator of the likelihood of severe trismus, providing a useful tool for early intervention and monitoring. These findings support the use of IID as an objective and reliable parameter for assessing the severity of trismus in clinical practice. Given the increased risk of severe trismus with the lingual approach, clinicians should carefully consider the potential benefits and risks of each surgical approach based on the patient's anatomy and the complexity of the impaction. The buccal approach may be preferable for reducing the risk of postoperative trismus, especially in cases of complex or mesioangular impactions. Yuan *et al.* (2021) [13] conducted a meta-analysis to compare the effects of lingual-based versus buccal-based mucoperiosteal flaps on postoperative morbidity following the surgical removal of impacted third molars. They found that the lingual-based flap was associated with better primary wound closure and specifically, the comma flap subtype showed superior outcomes in terms of postoperative pain, swelling and trismus when compared to the buccal-based flap. However, our study primarily focused on the impact of the surgical approach on the severity of trismus and we found that the lingual approach, despite potentially offering better wound closure, was associated with more severe trismus in the early postoperative period, particularly on Day 1 and Day 3. This contrast suggests that while the lingual-based flap may provide certain benefits in wound healing, it may also contribute to more immediate functional limitations such as trismus, which warrants further exploration of its impact on postoperative recovery. Based on the ROC analysis, IID on Day 1 can be used as a predictive marker for the severity of trismus. If the IID is below 24 mm, clinicians should initiate early intervention strategies, such as physiotherapy, anti-inflammatory medication and muscle relaxants, to mitigate the severity of trismus. Since pain and inflammation are closely linked to trismus, optimizing postoperative pain control through effective analgesia and managing facial swelling through cold compresses or corticosteroid administration may help reduce the incidence and severity of trismus. Previous studies have similarly identified the lingual approach as associated with a higher risk of postoperative complications such as trismus. A study by Hindy *et al.* (1995) [14] compared the modified lingual split technique with the conventional buccal approach in the surgical removal of impacted mandibular third molars. The study found that the lingual split technique was associated with a higher incidence of postoperative complications, including pain, edema and trismus, compared to the buccal approach. This suggests that the lingual approach may be linked to an increased risk of postoperative trismus. Our findings are in agreement with these studies, reinforcing the importance of minimizing tissue manipulation during third molar extraction. One limitation of this study is the lack of long-term follow-up to assess the persistence of trismus beyond the 7-day postoperative period, which could provide a more comprehensive understanding of the recovery trajectory. Future studies could explore the impact of different pain management protocols and physical therapy regimens on the severity and duration of trismus, as well as investigate the influence of patient-specific factors such as age and anatomical variations on surgical outcomes.

## Conclusion:

Patients undergoing mandibular third molar removal via the lingual approach are at significantly higher risk of postoperative trismus. Surgical approach should be chosen considering potential postoperative complications.

## Figures and Tables

**Table 1:** Comparison of surgical duration and postoperative trismus parameters between buccal and lingual approaches

**Parameter**	**Group A (Buccal)**	**Group B (Lingual)**	**p-value**
Mean Surgery Time (min)	28.5 ± 3.6	32.1 ± 4.1	0.002*
Pre-op IID (mm)	43.5 ± 2.5	42.8 ± 2.3	0.14
Post-op Day 1 IID	26.3 ± 4.1	20.5 ± 3.9	<0.001*
Post-op Day 3 IID	32.2 ± 3.8	26.4 ± 4.2	<0.001*
Post-op Day 7 IID	38.4 ± 3.1	34.1 ± 3.6	<0.001*
Severe Trismus (%)	10%	36%	<0.01*
OR (Lingual vs. Buccal)	-	5.1	95% CI: 2.0-12.8

**Table 2:** ROC curve analysis for predicting severe trismus using interincisal distance (IID) on Day 1

**Parameter**	**Value**
Area Under the Curve (AUC)	0.87 (95% CI: 0.79-0.94)
Optimal IID Cutoff (mm)	24 mm
Sensitivity	82%
Specificity	76%
